# Parental Perception of Remote Education in Pandemic: An Analysis Based on Children’s Cognitive Performance

**DOI:** 10.3390/children10101689

**Published:** 2023-10-16

**Authors:** Jasmin Bonilla-Santos, Alfredis González-Hernández, Dorian Yisela Cala-Martinez, Duvan Fernando Gómez Morales, Tatiana Padilla-García

**Affiliations:** 1 Department of Psychology, Universidad Cooperativa de Colombia, Street 11 No. 1-51, Neiva 410010, Colombia; dorian.calam@campusucc.edu.co; 2 Department of Psychology, Universidad Surcolombiana, Avenue Pastrana Borrero, Street 1, Neiva 410001, Colombia; alfredis.gonzalez@usco.edu.co (A.G.-H.); duvan.gomez@usco.edu.co (D.F.G.M.);

**Keywords:** executive function, motor skills, remote education, perception, COVID-19

## Abstract

The COVID-19 pandemic significantly changed patterns of human interaction, including in the educational sector, which was forced to transform relationships among students, families, and the academic community. The present study sought to establish the interrelationships between performance on cognitive tests during the preschool stage and the perceptions of parents about remote education in school children during the pandemic. The study included 100 preschool children from socially vulnerable sectors who underwent remote and distance learning in 2020 and 2021. The reliability of the applied questionnaire was determined through a confirmatory factor analysis. A structural equation model was constructed to determine the perceptions of parents about remote education based on cognitive performance during the preschool stage. The model fit yielded favorable results for predictive variables (*χ*^2^ = 7.734, DF = 9 [*p* = 0.561], the comparative goodness-of-fit index [CFI] = 1.000, root mean square error of approximation [RMSEA] = 0.000, standardized mean square residual [SRMR] = 0.069), and executive function (*χ*^2^ = 3.711, DF = 5 [*p* > 0.592], CFI = 1.000, RMSEA = 0.000, SRMR = 0.039) as latent variables that affected parents’ perceptions. These results indicate that parents’ perceptions of remote education are mediated by predictive aspects of learning and executive function during the preschool stage.

## 1. Introduction

The coronavirus disease 2019 (COVID-19) pandemic significantly changed patterns of human interaction, including in the educational sector, that forced the transformations of various aspects of relationships among students, families, and the academic community. These transformations were caused by the modification of conventional classes to the implementation of distance learning (both synchronous and asynchronously individual work separated from their social group and the evaluation model). These changes imposed an enormous challenge to meet new demands in the educational context, weakening support systems that are necessary for children to learn and develop [1], especially in the preschool stage because of the complex and multifaceted nature of the teaching–learning relationship [2].

Remote learning has proven to be beneficial in many aspects, but it also faces several challenges that can limit its optimal effectiveness [3]. Young children require direct supervision and accompaniment to enhance their academic skills, both of which are mediated by emotionally warm and responsive interactions [4], the quality of the supply of the educational system, and resources that are available at home during schooling [5]. This latter aspect has great relevance when considering that during school closures that were caused by mandatory social distancing during the pandemic, parents had to function as educators and tutors at home, providing guidance for learning, motivating, and monitoring the progress and development of their children’s schoolwork while performing their regular daily activities [6]. The perception of parents of the quality and support they receive during remote classes is mediated by multiple factors, such as the characteristics of the schools, support provided by teachers, the context, the family configuration, and the performance and physical and cognitive abilities of their children [7].

Cognitive ability plays a crucial role in assessing children’s development and exerts a significant influence on their behaviors and future socioeconomic achievements [8,9]. In the preschool stage, academic skills are subordinate to physical and cognitive development. Early childhood is considered a sensitive period at the level of central nervous system development, during which thinking, emotions, and socialization processes reach higher levels of complexity, allowing children to learn through exploration of the outside world [10]. Studies have shown that neuropsychological functions are potentiated from childhood [11], beginning with the development and strengthening of subcortical areas, including the reticular activating system, other areas of the brain stem, the thalamus, and inhibitory centers in the frontal cortex that allow the maintenance of alertness [12]. The development of these brain regions is followed by the maturation of regions that are associated with motor and sensory functions [13].

During the first years of life, motor activity promotes children’s intellectual, affective, and social development, favoring relationships with their environment [14]. Subsequently, the development of integrative and multimodal functions that are associated with tertiary sensory areas gives rise to such skills as reading, writing, and arithmetic [15]. The prefrontal cortex is associated with higher-order cognitive processes, such as executive function, and is the last brain area to fully mature [16,17]. Maturation of the prefrontal cortex demands a greater number of anatomical-physiological and environmental prerequisites for adequate performance.

Executive functions comprise control processes that regulate cognition and behavior [18]. There are three main executive functions: (1) response inhibition (i.e., the ability to inhibit dominant, automatic, or overbearing responses); (2) representation updating (i.e., the ability to monitor information to determine its relevance to a specific task and then update it appropriately by replacing old information with newer and more relevant information); and (3) alternation or flexibility (i.e., the ability to flexibly switch between tasks). Studies have identified an overlap between the requirements of initial basic education and skills that characterize executive functions, including oral language, which allow for the following of rules and the orientation of actions [19]. It is highly likely that distance learning imposes greater demands on children’s EF skills compared to traditional classroom education. This is due to the increased requirements of working memory associated with planning their own schoolwork. Additionally, it heightens the need for inhibition processes to avoid distractions at home, such as computer games and social media [20].

Neuropsychological functions are crucial to adjusting and adapting to changing environmental demands [17]. However, their emergence and development depend on biological factors and environmental experiences, implying that their developmental trajectory throughout life is sensitive to individual differences and the quality of contexts (e.g., home and school; Ref. [21]). Children who grow up in enriched environments with access to high-quality early education, who receive support from their parents, and who have the availability of resources for learning in the classroom and at home develop greater cognitive control and emotional regulation.

Conversely, children from disadvantaged socioeconomic backgrounds have a greater risk of lower cognitive control performance and slower developmental pathways [22] when considering low levels of parental education, little cognitive stimulation at home, limited linguistic interactions between parents and children, overcrowding, poor housing conditions, pre- and postnatal stress, and parents’ anxiety or depression [23,24]. The foregoing generates a greater burden for parents during homeschooling, where training processes are mediated by distance instructions, the need to apply technological tools, and greater parental involvement.

Research before the COVID-19 pandemic indicated that parents have mixed feelings about the remote learning experience, even under the best circumstances [25]. However, the pandemic exacerbated the challenges that can affect the participation of parents in remote learning environments, including low technical expertise to help their children access online learning and materials, a lack of internet access, or a slow learning pace of learners, and economic limitations about being able to purchase the required technological tools for remote education [26]. These factors further heighten existing pressures on parents [27], especially when considering that the pandemic caused job loss, permanent cohabitation with household members, fears of contagion, and high stress levels that limit quality interactions between parents and children during remote learning. Studies report that during the pandemic, low-income families tended to have less access to the internet and reliable devices compared to high-income families in the same city [28].

Although there are studies of the perceptions of parents about remote education in neurotypical populations such as those with cognitive disabilities [29,30], the impact of cognitive performance on positive and negative assessments of remote education has not been investigated. The present study analyzed relationships between variables that are considered facilitators of the learning process and perceptions of parents about distance education in schoolchildren in a situation of social vulnerability (i.e., the COVID-19 pandemic). Based on previous studies that indicated the existence of neuropsychological predictors of learning in early childhood that favor academic achievement in later school stages, we hypothesized that variables that are related to executive functions and predictors of learning impacted the perception of parents about distance education in schoolchildren during the pandemic.

## 2. Context

Latin America is considered one of the most unequal and economically unstable regions in the world [31]. This leads to educational, health, social, and cultural benefits that are available unequally among those who have resources and those who do not. In the educational field, children from poor families have been shown to be less likely to join and remain in the educational system, and they have high dropout rates [32]. Dropping out of school is associated with the need to generate income for the family and low expectations of education and school safety, in which the school is perceived as a hostile territory when considering the socioeconomic and cultural conditions of the environment [33]. 

In the Latin American country of Colombia, since 1960, there has been an upsurge of violence, causing inequities in access to the distribution of resources and a lack of guarantees for populations in peripheral areas [34]. The literature suggests that prolonged exposure to armed conflict, social deprivation, food insecurity, and limited access to education lead to negative cognitive outcomes in children. 

The educational sector in Colombia exemplifies a developing economy with high social inequity, considering the relatively high private spending by households that want access to a better education for their children and the existence of differences in the availability of resources between schools in the public and private sectors [35]. This scenario, combined with the COVID-19 pandemic, favored an increase in school absenteeism at the national level, which reached 13.7% in 2020 [36]. There is also a low coverage of, and sparse technological infrastructure for the development of, virtual and remote classrooms, as well as a decrease in family income due to the labor affectation of their parents because of the pandemic [37]. In 2020, an estimated 47.3% of households did not have internet access (74.3% did not have internet access in rural areas), and 58.4% did not have a computer or tablet to participate in the remote training process [38], thus limiting access to remote education.

## 3. Materials and Methods

A prospective cross-sectional design was used to investigate parents’ perceptions of the quality of remote education and their cognitive and emotional skills about the learning process of schoolchildren during the COVID-19 pandemic. Analyses of cognitive performance in the present study corresponded to the first cognitive assessment that was performed in 2019 before the COVID-19 pandemic. The sample included 100 first- and second-grade children from socially vulnerable sectors whose cognitive development was being monitored since preschool. Additionally, the sample was derived from the project “Characterization of neuropsychological predictors of learning problems in preschool children”, which described neuropsychological factors in 215 children (mean age = 5 years) who lived in four high-risk areas in the city of Neiva (classified as level 0, 1, and 2 in the Colombian stratification system, indicating low socioeconomic status). Due to the challenges of restrictive measures imposed by COVID-19, there was a loss of 50% of the sample (107 children). Specifically, out of the initial 107 participants, there were 75 children, and the educational institution lost telephone contact with some. Consequently, they could not be located. Eight students relocated from urban to rural areas and are currently not attending school. Additionally, 3 children migrated to the country’s capital. In one case, a child’s mother reported that the father took the child to another city, leading to their unavailability for the survey. Furthermore, 19 parents were engaged in informal work, making it difficult for them to establish communication due to time constraints. Lastly, 1 child’s mother chose not to participate in the survey.

The COVID-19 pandemic imposed economic challenges for many families in these localities, leading to school dropouts, a lack of access to technology, migration to rural areas, and changes in contact information. These challenges disrupted the initial neuropsychological characterization process and resulted in a sample loss of more than 50%. To understand the impact of remote education on children’s learning during the pandemic, the researchers contacted parents 1 and 2 years after the initial neuropsychological evaluation to gather their perceptions of remote education for their children. During 2020 and 2021, 20% of the children’s classes were performed remotely, and 80% were conducted asynchronously through workshop strategies and learning guides that were developed for teachers to be implemented by parents.

### 3.1. Participants

In the present study, the parents of preschool-aged students were recruited as participants. The mean age of the parents was 33.8 years (standard deviation [SD] = 9.3 years; range = 23–58 years). According to the Colombian educational system, the parents had completed their basic education (5 years of education; *n* = 15), secondary education with a bachelor’s degree (6 years of education; *n* = 52), a technical degree (8 years of education; *n* = 25), or university education (16 years of education; *n* = 7). In terms of occupation, 33 participants (33.6%) reported informal employment, 21 (21.4%) had a work contract, 9 (9.1%) had other work, and 35 (35.7%) reported no occupational activity.

### 3.2. Measures

*Neuropsychological Battery for Preschoolers* [39]. The objective of this test was to evaluate the normal and pathological courses of the neuropsychological development of 11 cognitive processes in the preschool stage (5 and 6 years old), including attention, memory, language, motor skills, and executive functions. Within the executive function domain, inhibition, working memory, cognitive flexibility, planning, abstraction, and risk–benefit processing were evaluated. This battery generated an index of the performance of children in cognitive processes that was evaluated for the preschool stage. Normalized total scores for each process were calculated, with a mean of 100 and SD of 15. The total score can be interpreted as the following: ≥116 (high normal performance), 85–115 (normal performance), 70–84 (mild to moderate impairment), and ≤69 (severe impairment).

*Remote Education Perception Questionnaire*

This questionnaire assessed three different aspects of parents’ perceptions of remote education. The first subscale consisted of four items that focused on family conditions and included questions about the family’s sociodemographic characteristics and economic aspects. The second subscale consisted of six items that measured parents’ perceptions of personal and pedagogical factors that are related to remote education, such as cognitive and emotional resources and the sufficiency of pedagogical strategies. The third subscale consisted of three items that examined the cognitive and emotional conditions of the child, including questions about their academic demands and cognitive abilities to respond to remote education. Each subscale included multiple items on a Likert scale, ranging from 0 to 4. For scale 1, the instrument allowed for a maximum score of 16 points and a minimum of 0. For scale 2, the participants could achieve a minimum score of 0 and a maximum of 24 points, and for scale 3, the range was from 0 to 12 points. 

A higher score indicated a more favorable perception of remote education. The items for the questionnaire were constructed by researchers who have extensive training and experience in psychology and neuropsychology in accordance with the specific needs of the project and context of the pandemic. The researchers carefully considered relevant factors that could affect the perceptions of remote education among parents of preschool children from socially vulnerable sectors. The final items were then validated through expert judgment to ensure their validity and reliability. An inter-rater agreement was measured by the Fleiss kappa index, which was 0.62, indicating substantial agreement. To select the final version with 13 items, the exploratory factor analysis and confirmatory factor analysis (CFA) were applied.

### 3.3. Procedure

The participants recruited for this study corresponded to the parents of students with a mean age of 33.8 years (SD: 9.3) (min. 23–max. 58) to whom the Questionnaire on Perception in Remote Education designed by the study researchers was given. The data were collected prospectively through the application of the questionnaire that evaluated the perception of remote education based on family characteristics, personal and pedagogical factors, as well as the cognitive and emotional condition of the child based on the neuropsychological characterization of preschool children with learning difficulties carried out during the year 2019.

### 3.4. Data Analysis

Descriptive variables were analyzed using means and SDs for quantitative data and percentages and frequencies for categorical variables. The exploratory analyses allowed the determination of whether there were extremes and missing data. Additionally, correlations among direct variables of the model were determined using the Pearson correlation coefficient. Structural equation modeling was used to test the hypotheses. A two-step procedure was used to investigate structural regression and mediation effects. The confirmatory factor analysis was used for latent variables. Goodness-of-fit statistics were calculated. For absolute fit, the *χ*^2^ statistic and degrees of freedom were calculated. For comparative fit, the comparative goodness-of-fit index (CFI), root mean square error of approximation (RMSEA), and the standardized mean square residual (SRMR) were calculated. Good model fit was reported to have a nonsignificant *χ*^2^ test result (*p* > 0.05), although this is rarely seen with large sample sizes. The CFI was ≥0.95. The RMSEA and SRMR were ≤0.05. The acceptable model fit was indexed with a CFI from 0.90 to 0.95. The RMSEA and SRMR were between 0.05 and 0.08. R studio 4.1.3 software was used for the data analysis. Structural equation modeling was conducted using the Lavaan package.

## 4. Results

The perception scale was interpreted domain by domain, in which higher scores indicated higher parental perceptions of remote education. The family condition score was 8.8 (SD = 1.3). The score for parental perceptions of personal and pedagogical factors for remote education was 14 (SD = 4.1). The score for parental perceptions of cognitive and emotional conditions of the child was 7 (SD = 2.5) (see Table 1).

### 4.1. Measurement Models (Confirmatory Factor Analysis)

**Figure 1:**
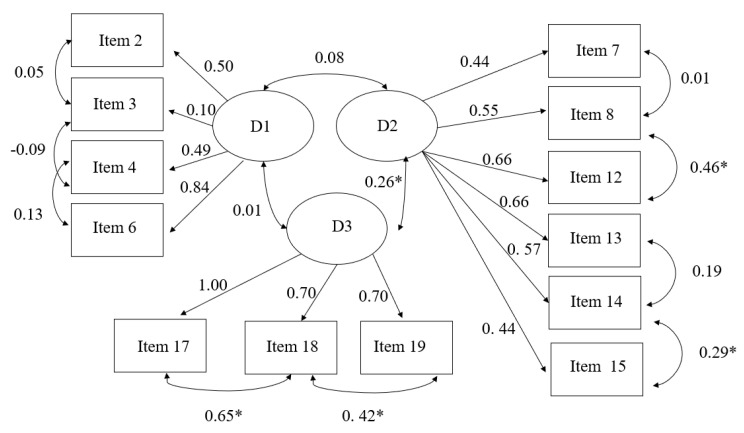
Confirmatory factor analysis of parental perception of remote education instruments. *χ*^2^ = 300.120 DF = 59 (*p* < 0.001), CFI = 0.80, RMSEA = 0.09, SRMR = 0.06. D1: family conditions; D2: parental perceptions of personal and pedagogical factors for remote education; D3: cognitive and emotional conditions of the child. * significant association.

*Remote Education Perception Questionnaire*. The CFA results for the perception questionnaire revealed that a three-factor model had a better fit (*χ*^2^ = 571.943, DF= 59 [*p* < 0.001], CFI = 0.80, RMSEA = 0.09, SRMR = 0.06) to explain the latent variable of parental perception. Items that had satisfactory factor loadings (between 0.34 and 0.83) for each of the three domains remained. Therefore, in domain 1, items 2, 3, 4, and 6 were selected. In domain 2, items 7, 8, 12, 13, 14, and 15 were selected. In domain 3, items 17, 18, and 19 were selected (Figure 1).

*Predictive variables of learning problems.* This latent variable was designed by the researchers from direct variables that were taken from subscales of the BANPE test, including motor skills, attention, recall memory, orientation, and language articulation. This variable was theoretically constructed and with the best fit (*χ*^2^ = 3.711, DF = 5 [*p* = 0.592], CFI = 1.000, RMSEA = 0.000, SRMR = 0.039).

*Executive functions.* The latent variable of executive functions comprised variables from subscales of the BANPE test, including inhibition, working memory, mental flexibility, planning, abstraction, and risk and benefit processing. The best fit indicated *χ*^2^ = 7.734, DF = 9 (*p* = 0.561), CFI = 1.000, RMSEA = 0.000, and SRMR = 0.069 (Table 2).

### 4.2. Theorical Model

**Figure 2:**
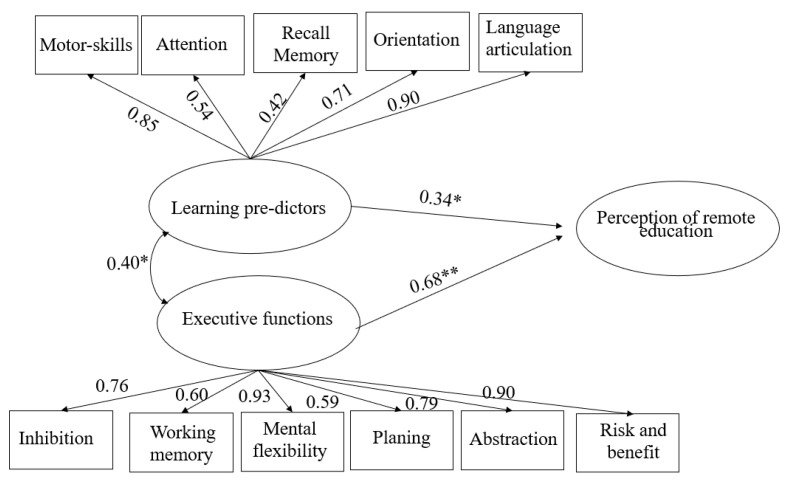
Theoretical structural equation modeling diagram. *χ*^2^ fit (DF) 55.123 (40) *p* = 0.056, CFI = 0.89, RMSEA = 0.10, SRMR = 0.064. * *p* < 0.05, ** *p* < 0.001.

In the present study, we proposed two hypotheses about relationships between latent variables. First, we hypothesized that the latent variable of learning predictors, which comprised motor skills, attention, recall memory, orientation, and language articulation, has a direct relationship with the latent variable of the perception of remote education, which comprises family conditions, parental perception, and cognitive and emotional conditions of the child. Second, we hypothesized that the latent variable of executive functions, which comprises inhibition, working memory, mental flexibility, planning, abstraction, and risk and benefit processing, has a direct relationship with the latent variable of the perception of remote education. By examining these hypothesized relationships, we sought to gain a better understanding of the factors that influence the perception of remote education among parents of children under conditions of social vulnerability. As hypothesized, a value of 0.68 suggested a positive moderately strong relationship between the executive functions and the perception of remote education, and a value of 0.34 suggested a positive low relationship between learning predictors and the perception of remote education (Figure 2).

For bivariate associations between the main variables of the study, we found a significant positive association between the performance of cognitive tasks (attention: *r* = 0.251, *p* < 0.005; working memory: *r* = 0.348, *p* < 0.005; planning: *r* = 0.292, *p* < 0.001) and parents’ perceptions of remote education in the time of the pandemic. Higher children’s competence in the preschool stage at the level of executive functions was associated with a greater probability that the parents’ perceptions of remote education in the time of the pandemic would be favorable. In turn, the associations between motor and cognitive skills were identified, including attention (*r* = 0.322, *p* < 0.001), recall memory (*r* = 0.279, *p* < 0.001), articulatory language (*r* = 0.205, *p* < 0.005), abstract thinking (*r* = 0.380, *p* < 0.001), and risk–benefit analysis (*r* = 0.432, *p* < 0.001). However, these associations were only low or moderate (Table 3).

## 5. Discussion

The present study analyzed the relationships between variables that are considered facilitators of the learning process and parents’ perceptions of distance education in schoolchildren under conditions of social vulnerability in the time of the COVID-19 pandemic. Based on cognitive performance in the preschool stage, a structural equation model was used to test the proposed hypothesis, showing that the performance of cognitive tasks that were associated with executive functions during preschool influenced parents’ perceptions of distance education during the pandemic. Distance learning implied greater demands at the level of executive function than traditional education in the classroom, attributable to greater demands on working memory that are associated with planning one’s own schoolwork.

It has been identified that difficulties in mastering executive function in the child population are an important factor in academic performance and imply a negative perception of parents about remote education because they imply greater demands from children in the context of academic activities, thus requiring more help from their parents to function adequately [40].

Likewise, the present results indicate a positive correlation between motor coordination and attention and performance of cognitive tasks that assessed articulatory language, working memory, abstract thinking, and risk–benefit analyses. Also, a relationship has been indicated between motor skills and cognition in children aged 4 to 16 years [41]. Similarly, it has been suggested that self-regulation and attention skills are necessary to engage in formal distance learning activities [42]. Parental education and emotional and sociodemographic conditions [7,43] are known to influence learning strategies among children who learn at home. In the present study, beyond the context of social vulnerability, the previous history in the development of executive functionality, planning, and working memory facilitated parents’ favorable perceptions of education at home. These findings are consistent with other studies where children’s cognitive ability was shown to influence parents’ perceptions of online learning [1,2]. 

The perception of the parents evaluated regarding the pedagogical strategies used in home education was deficient, possibly due to the lack of digital skills of the teachers, parents, and children, as well as less access to technological resources and the internet. It can be considered as a pedagogical teaching model but must be analyzed as strategies that had to be improvised by teachers to guarantee the right to education for children. These characteristics have been discussed in different studies [1]

Children with a low socioeconomic status are also more likely to have difficulties when adversity occurs, and the context of the COVID-19 pandemic was no exception. Adversity and uncertainty for families from vulnerable social sectors were common because of financial and social disadvantages, including restrictions of community support. Such adversity, uncertainty, and restrictions favored greater stress among family members, thus hampering quality interactions between parents, children, and the academic community that were necessary to support academic performance at home when teaching was conducted by household members [1,3]. The development and strengthening of cognitive skills are essential for adaptation to the educational context in its different modalities, allowing children and parents to feel less pressure and less reluctance to participate in virtual classrooms or distance learning.

The present results are especially applicable to preschool education. The results show that attention and cognitive functions, such as working memory and planning, prepare children to face and adapt to unexpected situations, such as the social isolation that was caused by COVID-19 and that forced alternative teaching methods to guarantee the right to education and favorable learning outcomes. It should be considered that under conditions of social vulnerability, the limited technological facilities, and the emotional, cognitive, economic, and family conditions, the parents influence the learning results obtained, and the cognitive aspects of children can also facilitate or interfere with these processes. The results have implications for educational institutions and government entities responsible for improving limited technological facilities to favor learning opportunities for children.

Educational institutions can adopt complementary actions that were favorable during the pandemic for children’s learning; in addition, the importance of schools communicating with parents about their children’s learning to promote learning at home and increase their participation and support in academic activities at home should be reiterated.

This exploratory study was conducted with a strict data analysis process to establish reliability by employing validity and reliability. However, some limitations must be considered. First, the relevant neuropsychological predictors that we used in the present study and the parental perception instrument were collected at different times (i.e., before the COVID-19 pandemic and after mandatory isolation), meaning that data collection closer to the initial assessment and during isolation could modify the results. Second, the study was conducted in public educational institutions with a relatively small number of participants. Third, the demography of our population was a group of children and parents from socially vulnerable sectors, which limits knowing the perspective of parents from the high socioeconomic strata. A deeper examination of public and private education systems about remote and online education during social isolation that was caused by the COVID-19 pandemic must be conducted to better generalize the results. It would also be interesting to contrast the perception of parents with teachers and children to allow a better understanding of education at home in the context of health uncertainty. In addition, the instrument used to evaluate the perception of the parents about remote education was designed to be applied quickly due to population characteristics such as access to the population, low level of education, and time availability of the parents. Therefore, it is recommended that psychometric adjustments are made for future applications.

## Figures and Tables

**Table 1:** Remote Education Perception Questionnaire.

Variable	Minimum	Maximum	Mean	SD
Family conditions (4 items)	0	16	8.8	1.3
Parental perceptions of personal and pedagogical factors for remote education (6 items)	0	24	14.7	4.1
Cognitive and emotional conditions of the child (3 items)	0	12	7	2.5

SD: standard deviation.

**Table 2:** Adjustment of indices for latent variables.

Latent Variables	*χ^2^* (DF)	CFI	RMSEA	SRMR
Perception of remote education	300.120 (59) *p* > 0.001	0.80	0.09	0.06
Learning predictors	3.711 (4) *p* = 0.446	1.000	0.000	0.039
Executive functions	7.734 (8) *p* = 0.458	1.000	0.000	0.069

Adequate model fit: *χ*^2^ test nonsignificant (*p* > 0.05), CFI ≥ 0.95, RMSEA and SRMR ≤ 0.05. Acceptable model fit: CFI = 0.90 to 0.95, RMSEA and SRMR 0.05–0.08.

**Table 3:** Correlations between observable variables.

Variable	1	2	3	4	5	6	7	8	9	10	11
1. Orientation	1										
2. Motor coordination	0.178	1									
3. Attention	0.199	0.322 **	1								
4. Recall memory	0.363 **	0.279 **	0.444 **	1							
5. Articulatory language	0.186	0.205*	0.267 *	0.326 **	1						
6. Inhibition	0.145	0.146	0.315 **	0.264 *	0.157	1					
7. Working memory	0.114	0.19	0.377 **	0.299 **	0.199 *	0.258 **	1				
8. Cognitive flexibility	−0.145	0.04	0.107	0.6	−0.067	−0.073	0.141	1			
9. Planning	0.208 *	0.118	0.312 *	0.314 *	0.187	0.305 **	0.440 **	0.086	1		
10. Abstract thinking	0.307 **	0.380 **	0.332 **	0.269 *	0.171	0.125	0.225 *	0.073	0.306 *	1	
11. Risk–benefit analysis	−0.15	0.432 **	0.182	0.061	0.136	0.015	−0.035	0.003	−0.125	0.022	1
12. Caregiver perception of remote education	0.023	−0.08	0.251 *	0.118	−0.056	0.123	0.348 *	0.12	0.292 **	0.097	−0.096

* *p* < 0.05, ** *p* < 0.001.

## Data Availability

Data supporting the findings of this study are available from the corresponding author upon reasonable request.

## References

[1] Pokhrel S., Chhetri R. (2021). A Literature Review on Impact of COVID-19 Pandemic on Teaching and Learning. High. Educ. Futur..

[2] Timmons K., Cooper A., Bozek E., Braund H. (2021). The Impacts of COVID-19 on Early Childhood Education: Capturing the Unique Challenges Associated with Remote Teaching and Learning in K-2. Early Child. Educ. J..

[3] Klosky J.V., Gazmararian J.A., Casimir O., Blake S.C. (2022). Effects of Remote Education During the COVID-19 Pandemic on Young Children’s Learning and Academic Behavior in Georgia: Perceptions of Parents and School Administrators. J. Sch. Health.

[4] Britto P.R., Lye S.J., Proulx K., Yousafzai A.K., Matthews S.G., Vaivada T., Perez-Escamilla R., Rao N., Ip P., Fernald L.C.H. (2017). Nurturing care: Promoting early childhood development. Lancet.

[5] Whitley J., Beauchamp M.H., Brown C. (2021). The impact of COVID-19 on the learning and achievement of vulnerable Canadian children and youth. Facets.

[6] Lagomarsino F., Coppola I., Parisi R., Rania N. (2020). Care tasks and new Routines for Italian families during the COVID-19 pandemic: Perspectives from women. Ital. Sociol. Rev..

[7] Ariyo E., Amurtiya M., Lydia O.Y., Oludare A., Ololade O., Taiwo A.P., Olukemi L.A., Ogunniyi D. (2022). Socio-demographic determinants of children home learning experiences during COVID-19 school closure. Int. J. Educ. Res. Open.

[8] Hu W., Mao Y., Huang K., Sun Y. (2022). Does Internet Entertainment Reduce the Cognitive Ability of Children? Evidence from the China Education Panel Survey. Behav. Sci..

[9] Zheng L., Weng Q., Gong X. (2021). Does preschool attendance affect the urban-rural cognition gap among middle school students? Evidence from China Education Panel Survey. J. Chin. Sociol..

[10] Meriem C., Khaoula M., Ghizlane C., Asmaa M.A., Ahmed A.O.T. (2020). Early Childhood Development (0–6 Years Old) from Healthy to Pathologic: A Review of the Literature. Open J. Med. Psychol..

[11] Mürner-Lavanchy I.M., Koenig J., Ando A., Henze R., Schell S., Resch F., Brunner R., Kaess M. (2020). Neuropsychological development in adolescents: Longitudinal associations with white matter microstructure. Dev. Cogn. Neurosci..

[12] Boen R., Ferschmann L., Vijayakumar N., Overbye K., Fjell A.M., Espeseth T., Tamnes C.K. (2021). Development of attention networks from childhood to young adulthood: A study of performance, intraindividual variability and cortical thickness. Cortex.

[13] Hadders-Algra M. (2018). Early human motor development: From variation to the ability to vary and adapt. Neurosci. Biobehav. Rev..

[14] Mas M., Jiménez L., Riera C. (2017). Systematization of the Psychomotor Activity and Cognitive Development. Educ. Psychol..

[15] Sutapa P., Pratama K.W., Rosly M.M., Ali S.K.S., Karakauki M. (2021). Improving Motor Skills in Early Childhood through Goal-Oriented Play Activity. Children.

[16] García L.F., Merchán A., Phillips-Silver J., González M.T.D. (2021). Neuropsychological Development of Cool and Hot Executive Functions Between 6 and 12 Years of Age: A Systematic Review. Front. Psychol..

[17] Treviño M., Beltrán-Navarro B., León R.M.-C.Y., Matute E. (2021). Clustering of neuropsychological traits of preschoolers. Sci. Rep..

[18] Miyake A., Friedman N.P. (2012). The Nature and Organization of Individual Differences in Executive Functions. Curr. Dir. Psychol. Sci..

[19] León C.B.R., Dias N.M., Martins G.L.L., Seabra A.G. (2018). Executive functions in preschool children: Development and relationships with language and behavior. Psicol. Teoria Prática.

[20] Thorell L.B., Fuermaier A.B.M., Christiansen H., Steinmayr R., Baeyens D., de la Peña A.G., Groom M.J., Idrees I., van der Oord S., Hoofdakker B.J.v.D. (2022). Distance learning during the COVID-19 pandemic for children with ADHD and/or ASD: A European multi-center study examining the role of executive function deficits and age. Child Adolesc. Psychiatry Ment. Health.

[21] Gottwald J.M., Achermann S., Marciszko C., Lindskog M., Gredebäck G. (2016). An Embodied Account of Early Executive-Function Development. Psychol. Sci..

[22] Last B.S., Lawson G.M., Breiner K., Steinberg L., Farah M.J. (2018). Childhood socioeconomic status and executive function in childhood and beyond. PLoS ONE.

[23] Lawson G.M., Hook C.J., Hackman D.A., Farah M.J. (2015). Socioeconomic status and the development of executive function: Behavioral and neuroscience approaches. Exec. Funct. Presch. Age Child. Integr. Meas. Neurodev. Transl. Res..

[24] Hackman D.A., Gallop R., Evans G.W., Farah M.J. (2015). Socioeconomic status and executive function: Developmental trajectories and mediation. Dev. Sci..

[25] Kong S.-C. (2017). Parents’ perceptions of e-learning in school education: Implications for the partnership between schools and parents. Technol. Pedagog. Educ..

[26] Ribeiro L.M., Cunha R.S., Andrade E Silva M.C., Carvalho M., Vital M.L. (2021). Parental Involvement during Pandemic Times: Challenges and Opportunities. Educ. Sci..

[27] Liu X., Zhao L., Su Y.-S. (2022). Impact of Parents’ Attitudes on Learning Ineffectiveness: The Mediating Role of Parental Self-Efficacy. Int. J. Environ. Res. Public Health.

[28] Francis D.V., Weller C.E. (2022). Economic Inequality, the Digital Divide, and Remote Learning during COVID-19. Rev. Black Political Econ..

[29] Ludji I., Marpaung T. (2021). Parents’ Perception on the Implementation of Home Learning during Covid-19. J. Basicedu.

[30] Aragón Mendizábal E., Mérida Serrano R., Serrano Díaz N. (2022). Percepción de las familias sobre el desempeño escolar durante el confinamiento por COVID-19. Comunicar.

[31] Mendez-Guerra C.A. (2015). Divergent and unequal development in Latin America: Causes and policy challenges. Globalization and Development Volume III: In Search of a New Development Paradigm.

[32] Fiszbein A., Stanton S. (2018). The Future of Education in Latin America and the Caribbean Possibilities for United States Investment and Engagement.

[33] Adelman M., Szekely M. (2016). School Dropout in Central America: An Overview of Trends, Causes, Consequences, and Promising Interventions. Policy Res. Work. Pap..

[34] Gordon E., Henao S.R., Duque A.Z., Dolan-Evans E. (2020). Power, poverty and peacebuilding: The violence that sustains inequalities and undermines peace in Colombia. Conflict Secur. Dev..

[35] Arbona A., Giménez V., López-Estrada S., Prior D. (2021). Efficiency and quality in Colombian education: An application of the metafrontier Malmquist-Luenberger productivity index. Socio-Econ. Plan. Sci..

[36] UNICEF (2021). Situación de las Familias con Niños, Niñas y Adolescentes en Colombia en Medio de la Crisis por COVID-19.

[37] Daza G.A.L., García C.F.G. (2020). Estado de excepción y restricción al derecho a la educación en Colombia por la COVID-19. Opinión Jurídica.

[38] Naciones Unidas de Colombia (2020). Análisis de Impacto Socioeconómico en la Crisis COVID-19. https://colombia.un.org/sites/default/files/2021-11/Analisis-de-Impacto-Socio-economico-en-la-crisis-COVID-19-sin-Prologo-VF_compressed1.pdf.

[39] Ostrosky Solís F., Lozano Gutierrez A., Gonzalez Osornio M.G. (2016). Batería Neuropsicológica Para Preescolares. Presentación. Edupsykhé.

[40] Crisci G., Mammarella I.C., Moscardino U.M.M., Roch M., Thorell L.B. (2021). Distance Learning Effects Among Italian Children and Parents During COVID-19 Related School Lockdown. Front. Psychiatry.

[41] Zeng N., Ayyub M., Sun H., Wen X., Xiang P., Gao Z. (2017). Effects of Physical Activity on Motor Skills and Cognitive Development in Early Childhood: A Systematic Review. BioMed. Res. Int..

[42] Barak M., Hussein-Farraj R., Dori Y.J. (2016). On-campus or online: Examining self-regulation and cognitive transfer skills in different learning settings. Int. J. Educ. Technol. High. Educ..

[43] Dong C., Cao S., Li H. (2020). Young children’s online learning during COVID-19 pandemic: Chinese parents’ beliefs and attitudes. Child. Youth Serv. Rev..

